# Multi-omics analysis reveals microbial and metabolic signatures associated with neutral detergent fiber digestibility in dairy cattle

**DOI:** 10.1186/s40104-026-01519-3

**Published:** 2026-11-06

**Authors:** Boyan Ma, Jianxin Xiao, Yiming Xu, Tianyu Chen, Shuai Liu, Shaodong Zou, Duo Gao, Yimin Zhuang, Mengmeng Li, Wei Wang, Shengli Li, Zhijun Cao

**Affiliations:** 1 State Key Laboratory of Animal Nutrition and Feeding, College of Animal Science and Technology, China Agricultural University, Beijing, 100193 China; 2 Animal Nutrition Institute, Sichuan Agricultural University, Chengdu, 611130 China; 3 Inner Mongolia Youran Dairy Co., Ltd., Hohhot, Inner Mongolia 010080 China; 4 National Technology Innovation Center for Dairy, Hohhot, Inner Mongolia 010080 China

**Keywords:** Dairy cattle, Metabolomics, Metagenomics, NDF digestibility, Rumen microbiome

## Abstract

**Background:**

The rumen microbiome is central to fiber degradation in dairy cows, yet the precise functional and metabolic networks driving variations in neutral detergent fiber (NDF) digestibility remain poorly understood. Elucidating these microbiome-host interactions is critical for improving forage utilization and advancing sustainable dairy production.

**Results:**

Employing an integrative metagenomics-metabolomics approach, we characterized the rumen microecology of lactating dairy cows exhibiting high and low NDF digestibility phenotypes (*n* = 10/group). Metagenomic profiling revealed a significant enrichment of fibrolytic taxa (*Prevotella* sp., Lachnospiraceae bacterium) and carbohydrate-active enzymes in the high-NDF group (HI). In addition, species-level differential abundance testing showed that *Methanobrevibacter* spp. was more abundant in the low-NDF group (LO) than in the HI group, whereas no archaeal differences were detected at the phylum or genus levels. Functional and metabolomic analyses indicated broader enrichment of amino acid degradation functions and higher concentrations of major volatile fatty acids (VFAs) in HI cows, consistent with stronger overall ruminal fermentation activity. Furthermore, VFAs and microbial composition explained 54.3% and 43.0% of the variation in NDF digestibility, respectively. An exploratory machine learning model based on selected annotated metabolite features showed moderate discriminatory performance, with an out-of-bag accuracy of 77.19%.

**Conclusions:**

Our findings suggest that coordinated shifts in rumen microbial composition, amino acid metabolism, and VFA production are associated with variation in NDF digestibility in dairy cattle. These results provide a basis for future studies aimed at improving fiber utilization through microbiome-informed nutritional or breeding strategies.

**Supplementary Information:**

The online version contains supplementary material available at https://doi.org/10.1186/s40104-026-01519-3.

## Background

Ruminants are pivotal to global food security, converting non-edible plant biomass into high-quality milk and meat for human consumption [1]. However, the sustainability of ruminant production is increasingly constrained by the efficient supply and utilization of feed [2]. In dairy ruminants, fiber digestibility is shaped by feed characteristics, rumen fermentation, and host-microbiome interactions, with the rumen microbiome playing a central role [3]. The rumen functions as a specialized bioreactor hosting a complex microbial ecosystem comprising bacteria, protozoa, and fungi that synergistically degrade fibrous materials [4, 5]. Recent advances in metagenomics have significantly expanded our understanding of this process, revealing functional specialization within the microbiome. For instance, *Ruminococcus* and *Fibrobacter* are classical cellulolytic genera in the rumen, whereas hemicellulose degradation is more commonly associated with genera such as *Prevotella* and *Butyrivibrio* [6]. Although less abundant, anaerobic fungi contribute disproportionately to the breakdown of recalcitrant lignocellulose through the secretion of diverse carbohydrate-active enzymes (CAZymes), including glycoside hydrolases (GH5, GH6, GH48), and the assembly of cellulosomes [7, 8]. Furthermore, metagenomics has enabled the recovery of genomes from uncultured taxa, uncovering a vast reservoir of novel CAZymes linked to fiber digestion efficiency in dairy cattle [9, 10].

Despite these advances, current strategies to improve neutral detergent fiber (NDF) digestibility have predominantly focused on exogenous feed additives [11], largely overlooking the intrinsic host-microbe metabolic interactions that drive inter-animal variation in fiber utilization. Recent studies indicate that rumen fiber degradation relies not only on the hydrolytic capacity of CAZymes but also heavily on complex metabolic cross-feeding within the microbiota [12–14]. However, how these micro-ecological metabolic networks synergistically regulate the host's fiber phenotype remains unknown. We hypothesize that the high NDF digestibility phenotype is not solely determined by the abundance of fibrolytic bacteria, but rather is driven by highly efficient energy and intermediate metabolite (specific VFAs and amino acid precursors) coupling networks between keystone taxa and the host. To test this, we employed an integrated metagenomic and metabolomic framework to characterize the microbial and metabolic signatures distinguishing dairy cows with high versus low NDF digestibility phenotypes.

This study aims to identify keystone taxa and functional pathways essential for fiber degradation and to elucidate the regulatory networks linking the rumen microbiome to variation in NDF digestibility. These insights will inform targeted strategies to enhance fiber utilization and promote sustainable ruminant production.

## Methods

### Animals’ management, sampling, and physiological parameters

The experimental procedures were approved by the Animal Care Committee at the China Agricultural University (Beijing, China) and were under the University’s guidelines for animal research (Approval No. AW82211202-1-1).

A total of 131 healthy multiparous Holstein dairy cows were collected from a commercial dairy farm in Gansu, China. These cows were housed in the same barn from the day of calving until 60 d postpartum and were used to measure the apparent digestibility of dietary nutrients. Total mixed ration (TMR) and fecal samples were collected from all experimental cattle twice daily (at 07:00 and 18:00) for the four days preceding the end of the experiment. The TMR samples were obtained using the five-point sampling method. Each composite sample was thoroughly mixed and then quartered to obtain a representative subsample of approximately 300 g for drying and subsequent analysis. Fecal samples were collected from each cow daily via gloved-hand rectal retrieval to avoid anal surface contamination. For each cow, repeated fecal collections obtained over the 4-day sampling period were composited to generate one representative sample for nutrient analysis; thus, the individual cow was considered the experimental unit for apparent digestibility. After collection, the total fecal output per cow was weighed, and a representative sample was created by combining equal quantities from each collection. This composite sample was then divided for different processing pathways: a 200-g aliquot was directly frozen at −20 °C, while an additional 80 g portion was acidified with 25 mL of 10% tartaric acid to stabilize nitrogenous compounds before storage at −20 °C. All feed and non-acidified fecal samples were dried at 65 °C, ground to pass through a 40-mesh sieve, and stored at room temperature. The acidified fecal samples were designated for the determination of crude protein (CP) content, whereas the non-acidified portions were used for analyses of other routine nutrients. The apparent digestibility of dietary nutrients was determined using the acid-insoluble ash indicator method as described by Block et al. [15], with acid-insoluble ash used as an internal marker in the TMR and fecal samples. The formula used for apparent digestibility calculation is provided in Additional file 1.

Based on the apparent digestibility measurements obtained from the whole herd, marked inter-animal variation in NDF digestibility was observed. To maximize phenotypic contrast for subsequent omics analyses, we selected 20 cows with extreme NDF digestibility values and retrospectively classified them into a high-NDF digestibility group (HI, 62.26% ± 1.25%) and a low-NDF digestibility group (LO, 44.16% ± 2.07%), which differed significantly (*P* < 0.01). Importantly, these cows were housed in the same pen and received the same batch of total mixed ration throughout the sampling period, thereby minimizing confounding from housing environment and basal diet. All cows were sampled within a narrow postpartum window (56–60 DIM), thereby minimizing variation due to stage of lactation. Additional animal characteristics, including milk yield during this period, body weight, and parity, are provided in Additional file 2. Because the cows were group-housed and fed a common TMR, individual dry matter intake (DMI) was not recorded at the animal level. The ingredient and nutrient composition of the early-lactation basal diet are provided in Additional file 3. Rumen fluid was collected from the 20 selected cows before the morning feeding for subsequent metagenomic and metabolomic analyses. Samples were collected via esophageal tubing with the first 50 mL discarded to avoid salivary contamination. Tubes were inserted to a depth of 1.5–2 m to ensure proper sampling of rumen contents, and the pH of the rumen liquid was measured immediately after collection. The rumen fluid was then filtered through four layers of gauze to remove large feed particles, and the resulting strained liquid fraction was aliquoted into 5-mL freezing tubes, snap-frozen in liquid nitrogen, and stored at −40℃ for subsequent analyses. Therefore, the metagenomic and metabolomic datasets generated in this study primarily represent the liquid-phase rumen fraction rather than the particle-associated solid fraction. The apparent digestibility of the dietary nutrients analyzed is shown in Additional file 2. Rumen VFA concentrations were determined using an Agilent 7890B-7000D gas chromatography platform following a method based on Erwin et al. [16] and Cao et al. [17], with detailed analytical procedures provided in Additional file 4.

### DNA extraction, metagenome sequencing, and sequence analysis

Total genomic DNA was extracted from the strained liquid-phase rumen samples using the FastDNA™ Spin Kit (MP Biomedicals, US) following the manufacturer’s instructions. The concentration and purity of the extracted DNA were determined using NanoDrop 2000 and TBS-380. The DNA quality was assessed on a 1% agarose gel. The DNA was fragmented to an average size of approximately 400 bp using Covaris M220 (Gene Company Limited, China) for paired-end library construction. The paired-end library was constructed using NEXTFLEX Rapid DNA-Seq (Bioo Scientific, Austin, TX, USA). Paired-end sequencing was performed on the Illumina NovaSeq (Illumina Inc., San Diego, CA, USA) at Majorbio Bio-Pharm Technology Co., Ltd. (Shanghai, China) using the NovaSeq 6000 S4 Reagent Kit v1.5 (300 cycles) according to the manufacturer’s instructions. Library construction, sequencing, and primary bioinformatic processing were conducted in collaboration with Majorbio Bio-Pharm Technology Co., Ltd. using its standard analytical workflow, and the resulting outputs were reviewed and verified by the authors before downstream interpretation.

The raw reads were filtered to exclude adapters and remove low-quality reads (length < 50 bp, or with a quality value < 20, or containing *N* bases) using fastp (version 0.20.0) [18]. Reads were aligned to the *Bos taurus* genome using BWA (version 0.7.9a) [19], and any read pairs where at least one end mapped to the host genome were removed to ensure strict host DNA depletion. The high-quality reads were then assembled into contigs using MEGAHIT (version 1.1.2) [20]. Contigs with lengths ≥ 300 bp were selected as the final assembly results and used for further gene prediction and annotation. Library construction, sequencing, and primary bioinformatic processing were conducted in collaboration with Majorbio Bio-Pharm Technology Co., Ltd. (Shanghai, China) using its standard analytical workflow, and the resulting outputs were reviewed and verified by the authors before downstream interpretation.

Open reading frames (ORFs) in contigs were identified using MetaGene (http://metagene.cb.k.u-tokyo.ac.jp/) [21]. A non-redundant gene catalog was constructed using CD-HIT (http://www.bioinformatics.org/cd-hit/, version 4.6.1) [22] with 95% sequence identity and 90% coverage. High-quality reads were aligned to the non-redundant gene catalogs to calculate gene abundance with 95% identity using SOAPaligner (http://soap.genomics.org.cn/, version 2.21) [23]. Representative sequences of the non-redundant gene catalog were annotated based on the NR database with an E-value cutoff of 1e-5 using Diamond (http://www.diamondsearch.org/index.php, version 0.8.35) [24] for taxonomic annotations. All taxonomic assignments, including archaeal reads, were retained in the taxonomic abundance tables. Because bacteria dominated the rumen metagenome, downstream functional interpretation focused primarily on the bacterial fraction; however, archaeal relative abundance was also compared between the HI and LO groups at the phylum, genus, and species levels using the same taxonomic comparison framework. KEGG annotation was conducted using Diamond (http://www.diamondsearch.org/index.php, version 0.8.35) against the Kyoto Encyclopedia of Genes and Genomes database (http://www.genome.jp/kegg/) with an E-value cutoff of 1e-5. Carbohydrate-active enzymes annotation was performed using hmmscan (http://hmmer.janelia.org/search/hmmscan) against the CAZy database (http://www.cazy.org/) [25] with an E-value cutoff of 1e-5.

The biological origins of the identified rumen metabolites were determined using the MetOrigin database [26], which classifies metabolites into host-derived, microbiota-derived, or co-metabolites (http://metorigin.met-bioinformatics.cn/), which classifies metabolites into host-derived, microbiota-derived, or co-metabolites. Functional enrichment analysis of differential metabolites was performed based on the KEGG pathway database to identify significantly enriched metabolic pathways [27, 28].

To visualize the "Taxonomy-Function-Metabolite" associations, we integrated metagenome sequencing data (Genus level), metagenomic functional prediction data (KEGG Orthology, KO), and metabolomic data. Sankey diagrams were constructed using the ggalluvial package in R to illustrate the flow of relative abundance from dominant microbial genera to specific functional genes (KOs) involved in "Valine, leucine and isoleucine biosynthesis" (ko00290) and "Histidine metabolism" (ko00340), and finally to the corresponding metabolites.

The relative abundance of functional genes (KOs) was normalized to counts per million (CPM). Differences in the abundance of key enzymes, including 3-isopropylmalate dehydratase small subunit (K01681) and histidinol-phosphate aminotransferase (K00817), between the High and Low groups were analyzed using Student's *t*-test (or Wilcoxon rank-sum test where appropriate). *P*-value < 0.05 was considered statistically significant.

Spearman rank correlation analysis was performed to evaluate the associations between the relative abundance of specific microbial genera (*Clostridium*) and metabolite concentrations (L-Histidine). Correlation coefficients (*r*) and *P*-values were calculated using the core test function in R. Relationships with |*r*| > 0.5 and *P* < 0.05 were considered strong and significant.

### Metabolomics profiling, identification, and analysis

The Liquid Chromatography–Tandem Mass Spectrometry analyses of the rumen metabolome were conducted on the Thermo UHPLC-Q Exactive HF-X system at Majorbio Bio-Pharm Technology Co., Ltd. (Shanghai, China). Data acquisition was performed using the Data Dependent Acquisition (DDA) mode. The raw data were imported into the Progenesis QI software (Waters, Milford, USA) for peak detection and alignment. The preprocessing generated a data matrix that consisted of retention time (RT), mass-to-charge ratio (*m/z*) values, and peak intensity. The data matrix was filtered such that metabolic features detected in at least 80% of any set of samples were retained. After filtering, minimum metabolite values were imputed for specific samples in which the metabolite levels fell below the lower limit of quantitation. Peak intensities were then normalized using the sum normalization method. Additionally, variables from quality control (QC) samples with a relative standard deviation (RSD) greater than 30% were excluded and log_10_ transformed to obtain the final data matrix for subsequent analysis. The accurate mass, MS/MS fragments, and isotope ratios were then matched against the HMDB (http://www.hmdb.ca/) and Metlin (https://metlin.scripps.edu/) databases to identify the metabolic features. The R package “ropls” (Version 1.6.2) was utilized to perform orthogonal partial least squares discriminant analysis (OPLS-DA). Differentially expressed metabolites (DEMs) were identified using univariate data analysis (*t*-test) with the following thresholds: variable importance in projection (VIP) > 1 and adjusted *P* value (FDR) < 0.05. The functions and metabolic pathways of differential metabolites were analyzed using the KEGG database.

A volcano plot was generated to visualize the distribution of differential metabolites based on log_2_ fold change (log_2_ FC) and −log_10_ *P*-values. Boxplots with jittered points were used to display the abundance distributions of nine selected differential metabolite features. Data visualization was performed using the "ggplot2" and "ggpubr" packages. The MetOrigin Online Analytics Platform was used to discriminate metabolites originating from the microbiome (http://metorigin.met-bioinformatics.cn/). This platform performs metabolic pathway enrichment analysis based on the original database and integrates the statistical correlations and biological relationships in the database using Sankey network visualization.

### Calculation of omics-explainability

The linear mixed model used for variance partitioning was specified as follows:

$${NDF}_{ijk}=\mu +DIM+Parity+Milk\;Yield+{Taxonomy}_{i}+{VFA}_{j}+{Metab}_{k}+Pen+Batch+{\epsilon}_{ijk}$$

where *NDF*_*ijk*_ is the observed NDF digestibility phenotype; *μ* is the overall mean; *DIM*, *Parity*, and *Milk Yield* represent the fixed effects. To account for potential collinearity among the omics layers, we modeled the omics features not as simple fixed effects, but as random effects based on their respective relationship matrices. Specifically, *Taxonomy*_*i*_, *VFA*_*j*_, and *Metab*_*k*_ represent the random effects for microbial composition, volatile fatty acids, and metabolites, respectively. These random effects are assumed to follow normal distributions based on their respective covariance matrices (*Taxonomy*_*i*_ ~ N(0, $\boldsymbol M\sigma_m^2$), where ***M*** is the microbial relationship matrix and $\sigma_m^2$ is the microbial variance). *Pen* and *Batch* are random effects to control for environmental variations, *ϵ*_*ijk*_ represents the residual error.

Crucially, by fitting these three highly correlated omics layers simultaneously in a joint Restricted Maximum Likelihood (REML) framework, the model estimates their conditional (independent) variance components by accounting for their shared covariance. The variance percentages reported subsequently represent their relative proportional contributions to the total omics-explained variance (which sum to 100%), rather than imply that they independently explain 100% of the absolute phenotypic variance.

The LMM analysis was performed using the lme4 package in R. The significance of each omics component was assessed using likelihood ratio tests (LRT), comparing the full model against a reduced model excluding the specific random effect. Multi-collinearity among explanatory variables was diagnosed using the variance inflation factor (VIF), ensuring all VIF values were below 5. Significance was declared at *P* < 0.05.

To explore the discriminatory potential of metabolite features associated with NDF digestibility, random forest (RF) classification was performed using the random Forest and rfUtilities (v2.1-5) packages [29]. Variable importance was assessed using Mean Decrease Accuracy (MDA), and model performance was summarized using the receiver operating characteristic (ROC) curve and out-of-bag (OOB) error rate. Given the limited sample size and the putative nature of untargeted metabolite annotations, the RF analysis was interpreted as exploratory rather than confirmatory. To elucidate associations between the rumen microbiota and metabolites, we employed Microbe-Metabolite Vectors (mmvec) [30] to model co-occurrence probabilities. The resulting neural network embeddings were visualized using the emperor plugin in QIIME 2 (v2020.8) [31].

### Statistical analysis

Cows were retrospectively classified into HI and LO groups according to extreme NDF digestibility values; therefore, all downstream analyses were conducted as phenotype-based two-group comparisons rather than as a randomized treatment trial. All statistical computations were performed in R (v4.x). Non-metric multidimensional scaling (NMDS) and analysis of similarity (ANOSIM) were conducted using the vegan package based on Bray–Curtis dissimilarities with 999 permutations. Phenotypic differences between groups were evaluated using Student’s *t*-test. Differential abundance of microbial taxa and functional features (Counts Per Million, CPM) was assessed using the Wilcoxon rank-sum test. *P*-values were adjusted for multiple testing using the Benjamini-Hochberg (BH) false discovery rate (FDR) method. Comparative analyses were restricted to taxa with a relative abundance > 0.1% and functional features with CPM > 5. Statistical significance was set at *P* < 0.05. All* P*-values reported in differential abundance and functional analyses throughout the text and corresponding figures represent BH-FDR adjusted Q-values unless otherwise stated.

To control for false positives in multiple comparisons across omics datasets, *P*-values were adjusted using the Benjamini–Hochberg false discovery rate (BH-FDR) method. Adjusted *P* < 0.05 was considered statistically significant, and effect sizes (Cohen’s d or Cliff’s delta) were reported for key differential features to evaluate the magnitude of differences alongside statistical significance.

## Results

### Nutrient apparent digestibility and VFA concentrations between the HI and LO groups

A significant difference in neutral detergent fiber (NDF) digestibility was found between the high-NDF digestibility group (HI) and the low-NDF digestibility group (LO); the HI group (62.26% ± 1.25%, mean ± SEM) was higher than the LO group (44.16% ± 2.07%) (*P* < 0.01). The crude protein (CP) digestibility in the HI group (74.32% ± 0.78%) was also higher than in the LO group (67.21 ± 1.05%, *P* < 0.01, Table 1). Regarding ruminal fermentation parameters, acetate (61.74 vs. 44.49 mmol/L), propionic acid (35.69 vs. 26.51 mmol/L), butyric acid (17.89 vs. 14.16 mmol/L), and isovaleric acid (1.80 vs. 1.45 mmol/L) were all higher in the HI group than in the LO group (*P* < 0.05, Table 1). Total VFA concentration was also higher in the HI group (120.42 vs. 89.60 mmol/L,* P* < 0.01), whereas NH_3_-N did not differ significantly between groups (8.53 vs. 7.79 mmol/L, *P* = 0.17). The acetate-to-propionate ratio was numerically similar between groups (1.73 in HI vs. 1.68 in LO), indicating that the higher VFA concentrations in the HI group reflected greater overall fermentation intensity rather than a marked shift toward propionate as the dominant end product. In addition, rumen pH tended to be lower in the LO group than in the HI group (6.21 vs. 6.47, *P* = 0.09; Table 1), although this difference did not reach statistical significance. Together, these results indicate a greater overall magnitude of ruminal fermentation in the HI group rather than a specific shift toward propionate.

**Table 1:** Nutrient apparent digestibility and rumen VFA concentrations in cows with high or low NDF digestibility

Items	HI (*n* = 10)	LO (*n* = 10)	SEM	*P *value
Nutrient apparent digestibility, % of DM				
NDF	62.26	44.16	2.37	< 0.01
CP	74.32	67.21	1.37	< 0.01
Milk yield, kg/d	72.89	64.84	1.44	< 0.01
Rumen pH value	6.47	6.21	0.16	0.09
Rumen fermentation parameters, mmol/L				
Acetate	61.74	44.49	3.19	< 0.01
Propionate	35.69	26.51	2.46	< 0.01
Isobutyrate	1.02	0.96	0.17	0.74
Butyrate	17.89	14.16	1.23	0.01
Isovalerate	1.80	1.45	0.19	0.03
Valerate	2.28	2.03	0.21	0.26
Total VFA	120.42	89.60	5.65	< 0.01
NH_3_-N	8.53	7.79	0.50	0.17

*DM* Dry matter, *NDF* Neutral detergent fiber, *CP* Crude protein, *VFA* Volatile fatty acid, *HI* High-NDF digestibility group, *LO *Low-NDF digestibility group

Peak lactation period = 60 ± 3 d

### Compositional profiles of rumen microbiome between the HI and LO groups based on metagenomics

**Fig. 1:**
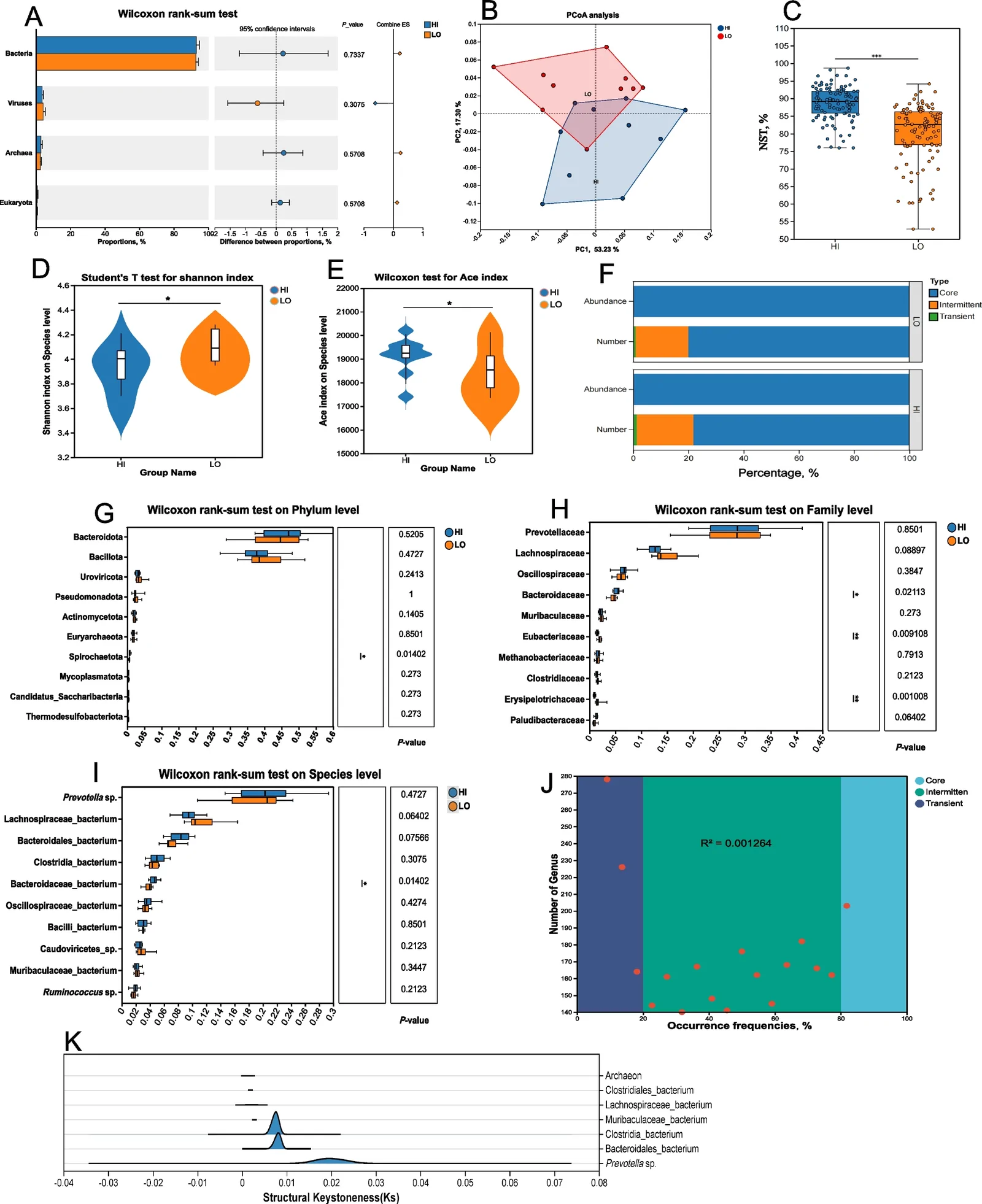
Microbial compositional profiles and diversity analysis of the HI and LO groups. **A** Comparison of microbial domains (Wilcoxon rank-sum test). **B** Principal coordinates analysis (PCoA) based on Bray–Curtis distances at the genus level; group differences assessed by ANOSIM. **C** Normalized stochasticity ratio (NST) analysis based on Jaccard distances at the species level. **D** and **E** Alpha diversity indices: Ace (**D**) and Shannon (**E**) (ANOVA; ^*^P < 0.05, ^***^P < 0.001). **F** Average relative abundance and proportion of transient, intermittent, and persistent (core) species and functions. **G**–**I** Differential abundance of bacteria and archaea at phylum, genus, and species levels (Wilcoxon rank-sum test; ^*^P < 0.05, ^**^P < 0.01). **J** Occurrence frequency versus relative abundance/number of species and functional genes. Red dots represent individual species or functions categorized as transient, intermittent, or core based on occurrence frequency. **K** Distribution of structural keystone values for the top 10 keystone species

The rumen metagenome was predominantly composed of Bacteria (92.66%), Viruses (3.82%), Archaea (2.83%), Eukaryota (0.68%), and Unclassified (0.01%) (Fig. 1A). Because bacteria constituted the vast majority of the rumen metagenome, downstream taxonomic description and functional interpretation focused primarily on the bacterial fraction. However, the archaeal fraction was retained in the taxonomic dataset and was also evaluated for between-group differences in relative abundance.

Distinct and significant differences in microbial diversity were observed between the HI and LO groups in both alpha and beta diversity indices (Fig. 1B–E). Principal coordinates analysis (PCoA) indicated clear clustering of the groups, highlighting differences in microbial community structure (Fig. 1B), supported by a significant difference in the normalized stochasticity ratio (NST, %) (Fig. 1C). Alpha diversity also exhibited significant disparities, with the Shannon index revealing notable differences (Fig. 1D) and the Ace index confirming variations in microbial richness (Fig. 1E).

The dominant bacterial phyla included Bacteroidota (45.12 ± 2.31), Bacillota (38.75 ± 2.04), and Pseudomonadota (2.56 ± 0.01) (Fig. S1A). The dominant bacterial genus was *Prevotella* (25.95 ± 1.41), unclassified Lachnospiraceae (10.68 ± 1.44), unclassified Bacteroidales (7.79 ± 0.89), and unclassified Clostridia (4.76 ± 0.44) (Fig. S1B). The dominant bacterial features at the species annotation level included *Prevotella* sp., *Lachnospiraceae bacterium*, *Bacteroidales bacterium*, and *Clostridia bacterium* (Fig. S1C), although several of these remained unresolved or uncultured at finer taxonomic resolution.

### Differences in bacteria and archaea between HI and LO groups based on metagenomics

At the phylum level, the abundance of Spirochaetota was significantly higher in the HI group than in the LO group (*P* = 0.01, Fig. 1G). At the family level, Bacteroidaceae was significantly enriched in the HI group (*P* < 0.01, Fig. 1H). At the species level, *Bacteroidaceae_bacterium* was one of the most significantly increased species in the HI group (*P* = 0.01, Fig. 1I). In total, 49 bacterial species differed significantly between the two groups. Among them, 16 species were enriched in the HI group, including 7 *Prevotella*-associated taxa, 2 *Bacteroidaceae-*related taxa, 2 *Succiniclasticum*-associated taxa, 1 *Kiritimatiella*-associated taxon, 1 *Treponema*-associated taxon, 1 *Alphaproteobacteria*-related taxon, 1 *Paludibacteraceae*-related taxon, and 1 unclassified taxon. (LDA > 2.0, *P* < 0.05). In contrast, 33 species were enriched in the LO group (LDA > 2.5, *P* < 0.05, Fig. S2A), indicating that the LO phenotype also possessed a distinct microbial signature rather than simply representing the absence of HI-associated taxa. For Archaea, the archaeal fraction was retained in the taxonomic analysis and evaluated separately based on relative abundance profiles. No significant between-group differences were detected at the phylum or genus levels. At the species level, formal differential abundance testing showed that *Methanobrevibacter* spp. (mainly s. *Methanobrevibacter boviskoreani*, *s. Methanobrevibacter oralis*, and *s. Methanobrevibacter* woesei) were significantly more abundant in the LO group than in the HI group (LDA > 2, *P* < 0.05, Fig. S2B). This pattern indicates that the archaeal difference observed here was compositional and species-level rather than a broad enrichment of the archaeal fraction across higher taxonomic ranks. *Persistent* (core) species dominated the microbial community based on their occurrence frequencies. The majority of data points were distributed within the intermediate frequency zone, while the number of species in the core and transient zones was relatively low. The distribution pattern exhibited a poor fit to the neutral model (*R*^2^ = 0.001264; Fig. 1L). The proportional abundance and number of persistent (core) species were significantly higher than those of transient and intermittent types in both the LO and HI groups (Fig. 1F). The top 10 structural keystone species (including *s_archaeon* and *s_Clostridiales_bacterium*) were identified based on network topology, and their structural keystone index (Ks) distribution is shown in Fig. 1M. Because this analysis was performed at the community-network level rather than as a group-specific differential network comparison, these taxa were interpreted as indicators of overall community structure rather than direct drivers of the HI/LO divergence.

### Functionals of rumen microbiome between the HI and LO groups based on metagenomics

**Fig. 2:**
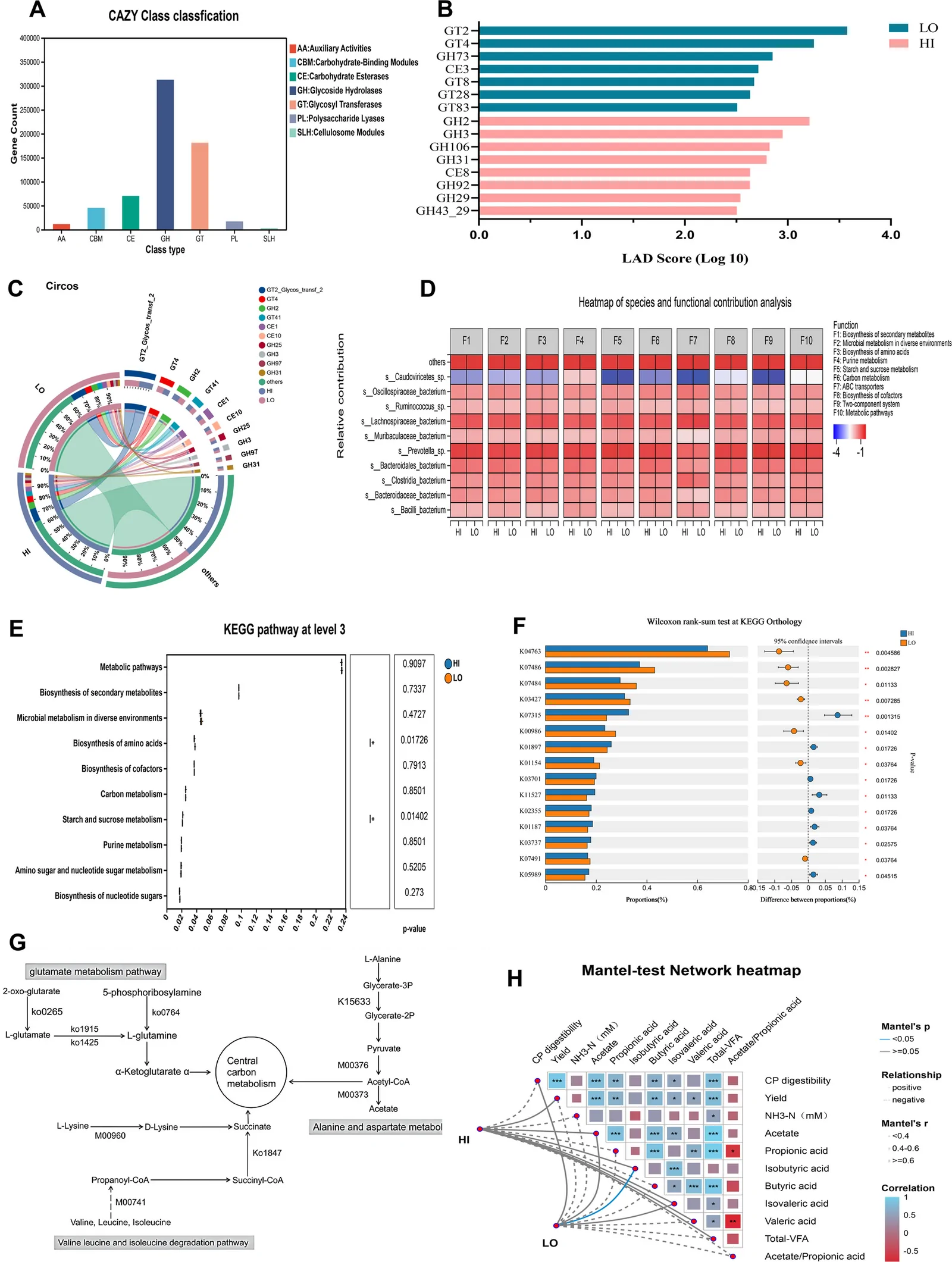
Microbial functional differences between the HI and LO groups. **A** Distribution of gene counts across seven carbohydrate-active enzyme (CAZy) classes. **B** LEfSe analysis identifying differentially abundant CAZymes (LDA score indicates effect size). **C** Circos plot illustrating associations among core CAZyme gene families. Line thickness is proportional to association strength. **D** Heatmap showing the relative contribution of the top 11 bacterial phyla to the top 10 core CAZyme families (F1–F10). Color gradient indicates the contribution degree (blue: low; red: high). **E** and **F** Differentially enriched KEGG modules at level 3 pathways (Wilcoxon rank-sum test; ^*^P < 0.05, ^**^P < 0.01). **G** Differential metabolic pathways, molecules, and KOs identified between groups. **H** Mantel test network heatmap linking microbial communities (HI: red; LO: blue) to environmental factors. Line width corresponds to Mantel’s *r* statistic, with solid and dashed lines indicating positive and negative correlations, respectively. The heatmap displays pairwise Spearman correlations between environmental factors (^*^P < 0.05, ^**^P < 0.01)

A total of 630 genes encoding carbohydrate-active enzymes (CAZymes) were identified, which belonged to 25 auxiliary activities (AAs), 81 carbohydrate-binding modules (CBMs), 16 carbohydrate esterases (CEs), 296 glycoside hydrolases (GHs), 111 glycosyltransferases (GTs), and 101 polysaccharide lyases (PLs). Among these, genes encoding GT2 (9.20 ± 0.55) were the most dominant, followed by those encoding GH2 (5.80 ± 0.23), GT4 (4.67 ± 0.28), and GH3 (1.82 ± 0.13) (Additional file 5, Fig. 2A). When comparing identified CAZymes, a total of 15 family-level enzymes were significantly different between the two groups based on LEfSe analysis. Among these, 8 family-level enzymes (7 GH and 1 CE) were significantly enriched in the HI group, while 7 family-level enzymes (1 GH, 5 GT, and 1 CE) were significantly enriched in the rumen of the LO group (LDA > 2.5, *P* < 0.05, Fig. 2B). The CAZyme families enriched in the HI group included GH2, GH3, GH106, GH31, GH92, GH29, GH43-29, and CE8. Functionally, these families are more consistent with pectinolytic, debranching, and oligosaccharide-directed activities than with canonical endo-acting cellulase or xylanase systems. In contrast, the LO group was characterized by enrichment of GT2, GT4, GH73, CE3, GT8, GT28, and GT83, indicating a functional profile with a greater representation of glycosyltransferase-related activities than that observed in the HI group. A sample-species relationship Circos plot revealed distinct community compositions between the HI and LO groups (Fig. 2C). The ribbon connections illustrated associations between specific dominant species and sample groups, indicating a higher relative abundance of certain species in the HI group. Furthermore, co-occurrence patterns among core CAZyme families (GT2, GT4, GH2, GT41) were identified using a Circos plot, with thicker connecting lines indicating stronger ecological links between gene families.

Significant differences were observed in the contributions of different phyla to CAZyme functions at the family level (Fig. 2D). Specifically, Bacteroidota contributed highly to several CAZyme families (F1, F3, F5), while Bacillota and Actinobacteriota made substantial and distinct contributions to other functions (F2, F8), respectively. This analysis identified the core taxonomic groups driving key carbohydrate metabolic functions when comparing HI and LO groups.

For the KEGG profiles, a total of 641 endogenous third-level pathways were observed (Additional file 6). These pathways belonged to six level-1 categories, including Metabolism (66.38 ± 0.49), Cellular Processes (12.93 ± 0.18), Environmental Information (7.06 ± 0.07), Genetic Information (3.35 ± 0.01), and Organismal Systems (4.21 ± 0.16). We further identified 48 level-2 key pathways, including Global and overview maps (31.04 ± 0.25), Carbohydrate metabolism (10.62 ± 0.10), Amino acid metabolism (7.08 ± 0.09), Glycan biosynthesis and metabolism (5.80 ± 0.03), Metabolism of cofactors and vitamins (5.84 ± 0.06), and Energy metabolism (4.74 ± 0.05). When comparing the identified KEGG pathways, no differences were observed in any of the detected KEGG pathways at level 1 (*P* > 0.05), while amino acid metabolism was significantly different between the two groups at level 2 (*P* < 0.05). The analysis of differences in amino acid metabolism at the KEGG level 3 showed that the alanine aspartate, glutamate metabolism, as well as the valine leucine and isoleucine degradation pathways, were significantly different between the two groups (*P* < 0.05, Fig. 2E). Furthermore, four modules (M00373, M00741, M00376, M00960) (*P* < 0.05, Fig. 2F) and six downstream pathways (K01915: glnA, GLUL, glutamine synthetase; K00265: gltB, glutamate synthase (NADPH) large chain; K00764: purF, PPAT, amidophosphoribosyltransferase; K15633: gpmI, 2,3-bisphosphoglycerate-independent phosphoglycerate mutase; K01847: MUT, methylmalonyl-CoA mutase; K01425: glsA, GLS, glutaminase) were enriched in the HI group (Additional file 7 and Additional file 8). As summarized in Fig. 2G, the differential analysis suggested that several amino acid degradation pathways were functionally associated with central carbon metabolism, including glutamate metabolism, alanine and aspartate metabolism, and valine, leucine, and isoleucine degradation. These pathway-level associations involved intermediates such as pyruvate, alpha-ketoglutarate, and succinyl-CoA-related reactions, and may reflect coordinated metabolic functions associated with fermentative processes in the HI group. Significant correlation patterns were observed between the overall community structures of the HI and LO groups and the measured environmental factors (Fig. 2H). Specifically, the community structure showed significant correlations, either positive or negative, with certain key environmental factors (CP digestibility, NDF digestibility, Mantel's *r*, *P* < 0.05). Furthermore, the heatmap on the right illustrates a complex network of interactions among these environmental factors themselves.

### Rumen metabolome profiling differed between HI and LO groups

**Fig. 3:**
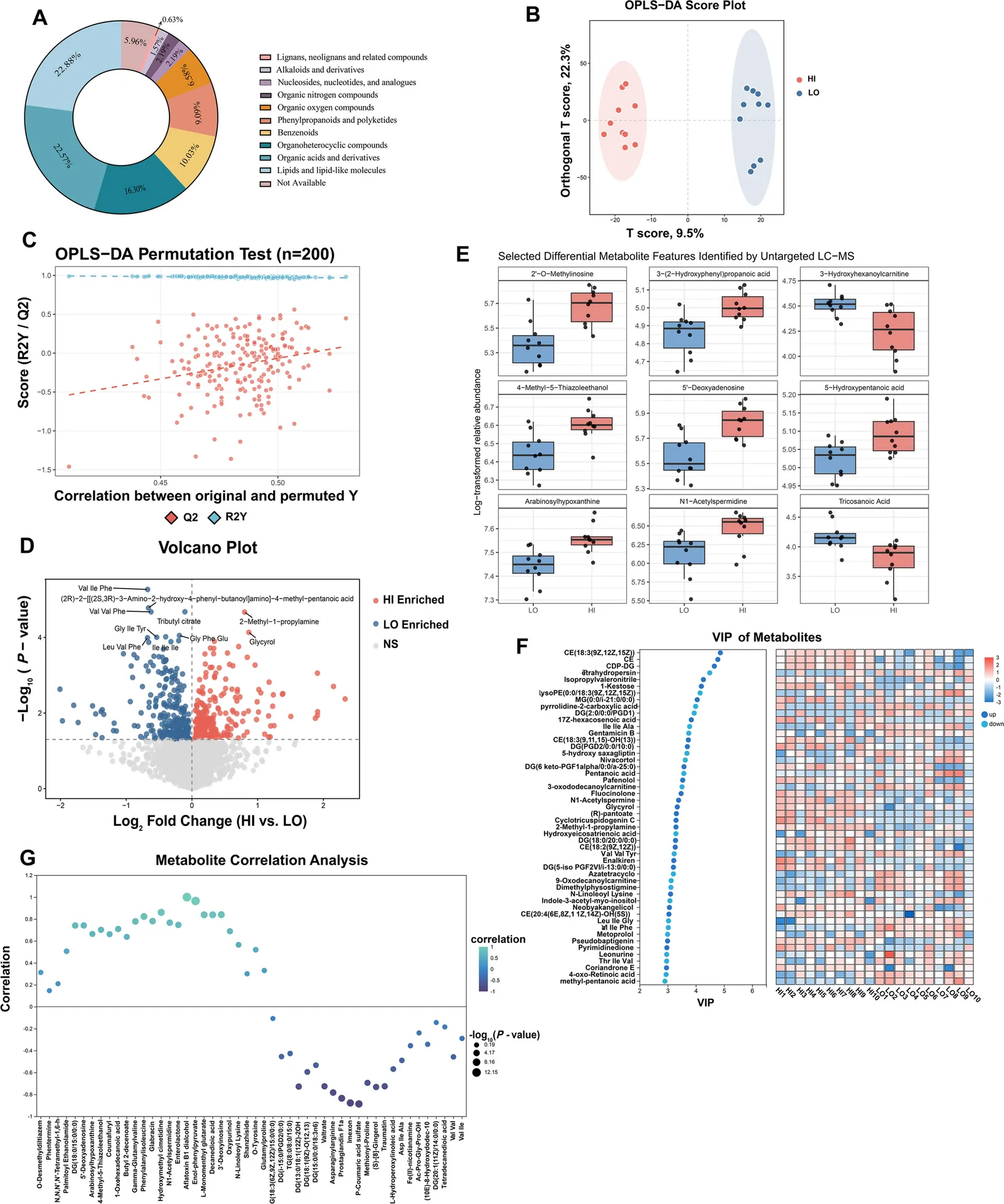
Differences in rumen metabolome profiles between HI and LO groups. **A** Classification of identified metabolites based on HMDB superclass. **B** OPLS-DA score plot showing distinct separation between groups. **C** Permutation test (*n* = 200) validating the robustness of the OPLS-DA model. **D** Volcano plot of differential metabolites (VIP > 1, *P* < 0.05). Red and blue points represent significantly upregulated and downregulated metabolites in the HI group, respectively. **E** Boxplots of nine selected differential metabolite features identified by untargeted LC-MSAHPBA-MPA: 2(R)-2-((2S,3R)-3-Amino-2-hydroxy-4-phenylbutanoyl) amino]-4-methylpentanoic acid. **F** KEGG pathway enrichment analysis of the differential metabolites. **G** Spearman correlation analysis of the differential metabolites. Dot color indicates the Spearman correlation coefficient, and dot size indicates the −log_10_(*P*-value)

A total of 434 metabolites were identified in the ruminal contents, among which 319 metabolites were annotated in the Human Metabolome Database (HMDB). Classification analysis revealed that the metabolome was dominated by "Lipids and lipid-like molecules" (22.88%) and "Organic acids and derivatives" (22.57%), followed by "Organo heterocyclic compounds" (16.30%), "Benzenoids" (10.03%), and "Phenylpropanoids and polyketides" (9.09%) (Fig. 3A).

A clear separation between the High (HI) and Low (LO) NDF digestibility groups was observed, with all samples falling within the 95% Hotelling's T2 ellipse (Fig. 3B). The robustness of the model was validated using a permutation test (*n* = 200), which confirmed that the observed separation was statistically significant and not due to overfitting (Q^2^ > 0.5, intercept < 0; Fig. 3C).

The volcano plot visualized the global differential metabolite pattern, revealing distinct clusters of upregulated and downregulated features (Fig. 3D). We further selected nine differential metabolite features for visualization using boxplots (Fig. 3E). Because these annotations were derived from untargeted LC–MS and were not validated with authentic standards, they should be interpreted as putative metabolite assignments rather than confirmed biomarkers. Nevertheless, these selected features illustrated a clear metabolic distinction between the HI and LO groups, indicating that differences in NDF digestibility were accompanied by substantial remodeling of the ruminal metabolic profile. The differential metabolites were significantly enriched in specific metabolic pathways, primarily including Amino acid metabolism (Valine, leucine and isoleucine biosynthesis; Histidine metabolism) and Metabolism of cofactors and vitamins (Fig. 3F). Consistent with this, a broader classification of the top 30 differential metabolites showed that the majority belonged to amino acid metabolism (33.97%), followed by lipid metabolism (11.32%) and cofactor/vitamin metabolism (11.32%) (Additional file 9).

Significant associations were observed between differential metabolic features and specific functional sets, suggesting possible links between the rumen metabolome and variation in NDF digestibility (Fig. 3G).

### Multi-omics analysis reveals microbial contribution to amino acid biosynthesis in the rumen

**Fig. 4:**
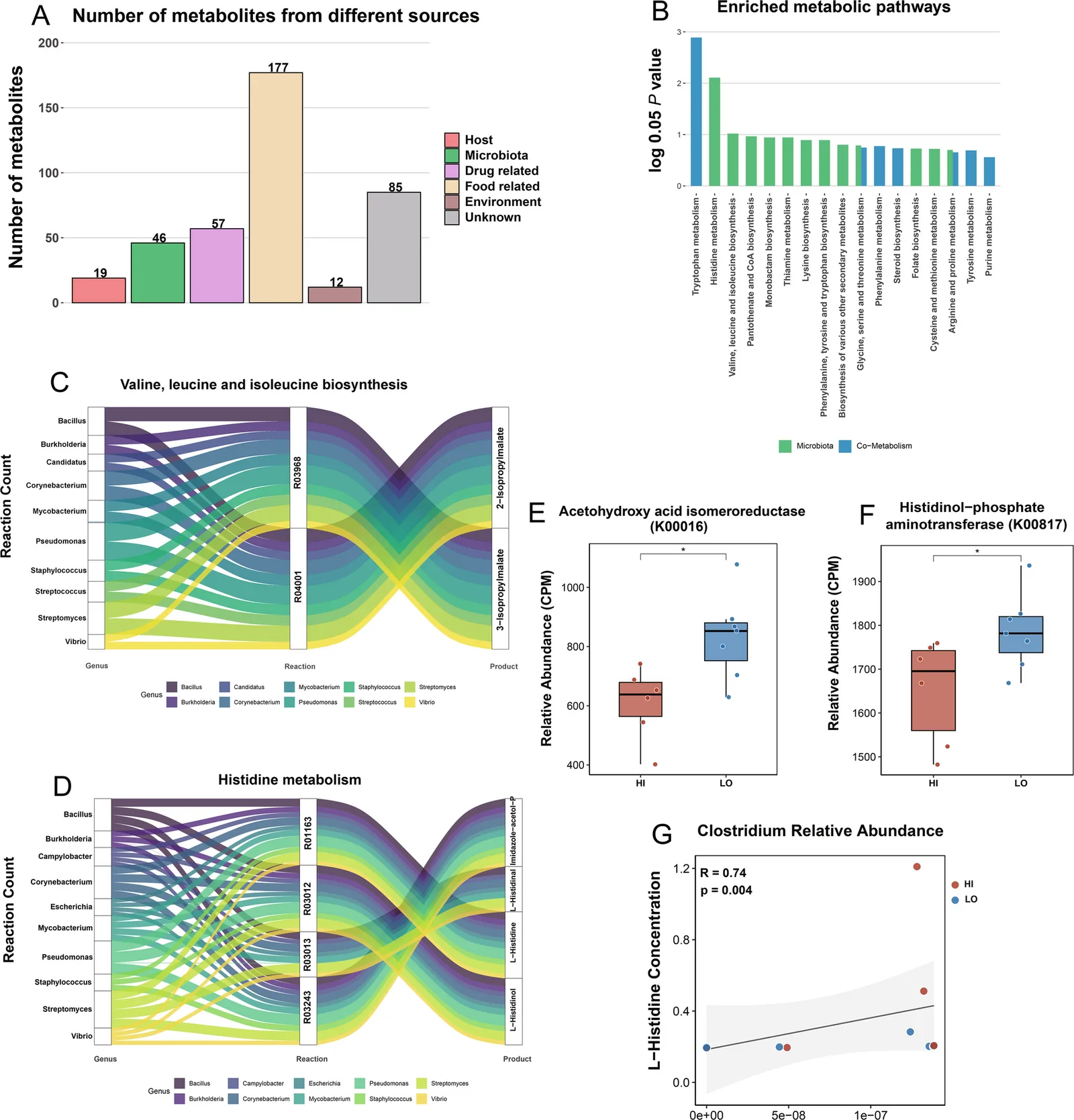
Integrative analysis of metabolite origins and functional pathways. **A** Classification of metabolites based on their origin (Host, Microbiota, or Co-metabolism) using MetOrigin analysis. **B** KEGG pathway enrichment analysis of microbiota-derived and co-metabolites. **C** and** D** Sankey diagrams tracing multi-omic associations in (**C**) Valine, leucine and isoleucine biosynthesis (map00290) and (**D**) Histidine metabolism (map00340) pathways. Flows represent the contribution of bacterial species to key enzymes (K00016, K00817) and subsequent metabolites. **E** and** F** Relative abundance (CPM) of (**E**) L-lactate dehydrogenase (K00016) and (**F**) histidinol-phosphate aminotransferase (K00817) between groups (^**^P < 0.01, Student's *t*-test). **G** Spearman correlation between *Clostridium* abundance and L-Histidine concentration. Shaded area represents the 95% confidence interval

A substantial proportion of the annotated metabolic features were classified by MetOrigin as co-metabolites or microbiota-associated compounds (Fig. 4A). Based on these annotations, pathway enrichment analysis suggested perturbations in amino acid metabolism, particularly in valine, leucine and isoleucine biosynthesis (KEGG map00290) and histidine metabolism (KEGG map00340) (Fig. 4B). Notably, these biosynthesis-associated signatures were relatively more prominent in the LO group. These results should be interpreted cautiously because the metabolite annotations were not validated with authentic standards. Sankey diagrams integrating taxonomic, functional, and metabolic data illustrated associations among annotated bacterial taxa, selected functional genes, and downstream metabolite features in the branched-chain amino acid and histidine pathways (Fig. 4C and D). Because many dominant taxa were unresolved at finer taxonomic levels, these Sankey plots should be interpreted as pathway-level association patterns rather than direct taxon-to-metabolite mechanistic links. After verification against KEGG, K00016 was identified as L-lactate dehydrogenase rather than acetohydroxy acid isomeroreductase. The relative abundance of K00016 was significantly higher in the LO group than in the HI group (*P* < 0.01, Fig. 4E), indicating a stronger lactate-associated functional signal in the LO group. Consistently, the abundance of K00817, a key enzyme in histidine biosynthesis, was also significantly lower in the HI group than in the LO group (*P* < 0.01, Fig. 4F), further suggesting that the LO phenotype retained stronger amino acid biosynthesis-associated functions. A specific *Clostridium* taxon exhibited a strong positive correlation with fecal L-histidine concentrations (*r* > 0.8, *P* < 0.001, Fig. 4G). This finding implies that the depletion of this specific *Clostridium* member in the High group may directly contribute to the observed reduction in L-histidine levels, providing a potential target for microbiota-based intervention.

### Integrative analysis and predictive modeling of NDF digestibility

**Fig. 5:**
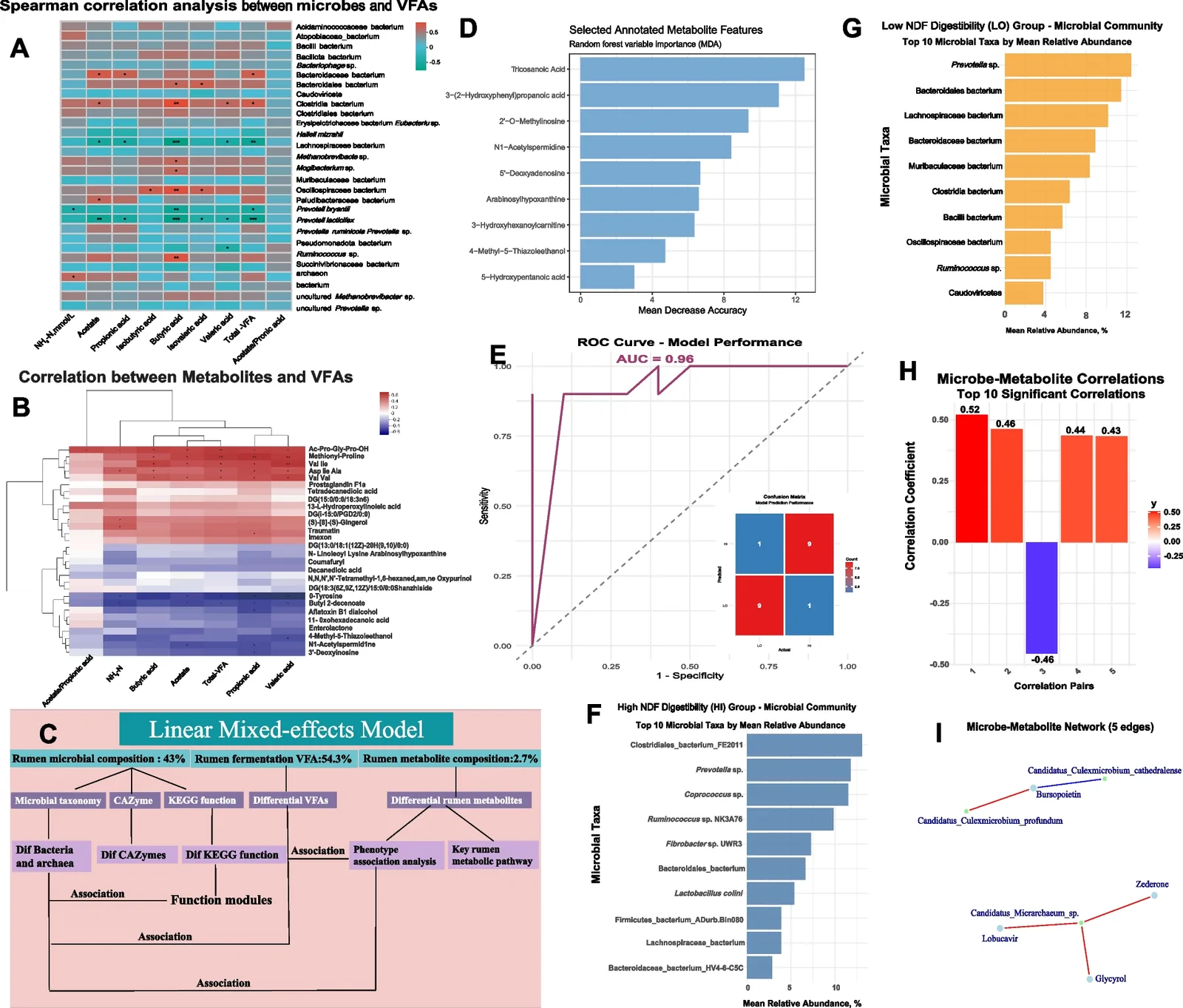
Multi-omics integration and predictive modeling of NDF digestibility. **A** and **B** Heatmaps of Spearman correlations between volatile fatty acids (VFAs) and rumen metabolites (**A**) or microbial taxa (**B**). **C** Variance partitioning analysis (VPA) quantifying the contribution of rumen microbial composition, VFA profiles, and the metabolome to NDF digestibility variance (linear mixed-effects model). **D** Top 20 annotated metabolite features associated with NDF digestibility ranked by Mean Decrease Accuracy (MDA) in the random forest model. **E** Receiver operating characteristic (ROC) curve for the exploratory random forest model distinguishing high (HI) and low (LO) NDF digestibility groups. Insets show the training-set area under the curve (AUC), the confusion matrix, and the corresponding out-of-bag classification performance described in the text. **F** and **G** Mean relative abundance of the top 10 microbial species enriched in the HI (**F**) and LO (**G**) groups. **H** Significant microbe-metabolite correlation pairs (Spearman’s rank correlation; *P* < 0.1, |*r*| > 0.4). **I** Network visualization of five key microbe-metabolite interactions distinguishing HI and LO phenotypes

Distinct microbial and metabolic signatures were associated with ruminal VFA profiles based on Spearman correlation analysis. Several microbial taxa, including Bacteroidaceae bacterium, Bacteroidales bacterium, Clostridia bacterium, Oscillospiraceae bacterium, *Ruminococcus* sp., unclassified archaeon, *Methanobrevibacter* sp., *Mogibacterium* sp., and Paludibacteraceae bacterium, were positively correlated, while *Hallella mizrahii*, *Prevotella bryantii*, *Prevotella lacticifex*, and Pseudomonadota bacterium were negatively correlated with ruminal VFA (Fig. 5A). This pattern suggests that a consortium of fibrolytic bacteria and archaea is synergistically linked to enhanced fermentative activity. Parallel analysis of rumen metabolites identified several metabolites that were positively correlated with rumen VFA, such as Ala-Asp-Ile, AC-Pro-Gly-Pro-OH, (S)-[8]-Gingerol, Methionyl-Proline, H-Val-Val-OH, and H-Val-Ile-OH, while Shanzhiside and 4-Methyl-5-Thiazoleethanol were negatively correlated (Fig. 5B). This implies that protein degradation products may be intertwined with the microbial metabolic network driving VFA production.

Rumen fermentation VFA profiles accounted for the largest proportional share (54.3% ± 9.2% SE) of the total omics-explained variance (Fig. 5C), underscoring their paramount importance. Rumen microbial composition explained 43.0% (± 8.7% SE) of the omics-explained variance, highlighting its strong direct and indirect influence. In contrast, the rumen metabolome alone accounted for a modest 2.7% of the variance, suggesting that its primary role may be mediated through its interaction with microbes and fermentation outputs.

A random forest model was further used to evaluate the discriminatory contribution of selected annotated metabolite features associated with NDF digestibility (Fig. 5D). In the training set, the exploratory model achieved an area under the curve (AUC) of 0.96 in the ROC analysis (Fig. 5E). However, the out-of-bag (OOB) error rate was 0.2281, corresponding to an accuracy of 77.19%, with 8 of the 10 HI cows and 7 of the 10 LO cows correctly classified. These results indicate that the selected metabolite features had preliminary discriminatory value, although the limited sample size and the putative nature of untargeted metabolite annotations warrant cautious interpretation. Microbial community analysis revealed distinct composition patterns between NDF digestibility groups. The HI group showed higher abundance of taxa such as *Clostridium* sp., *Prevotella* sp., *Fibrobacter* sp., and related organisms (Fig. 5F), whereas the LO group displayed its own enriched profile, including *Propionibacterium* sp., *Acidipropionibacterium* sp., *Thermoanaerobacter* sp., and other taxa (Fig. 5G). Thus, the contrast between HI and LO groups reflected two different microbial configurations rather than a one-sided gain of taxa in the HI group alone. Network analysis identified significant microbe-metabolite correlations (Fig. 5H and I) associated with the two phenotypes, with 5 significant interaction pairs identified through Spearman correlation analysis (FDR-adjusted *P* < 0.1, |*r*| > 0.4). These results indicate that the selected metabolite features had preliminary discriminatory value, although the limited sample size and the putative nature of untargeted metabolite annotations warrant cautious interpretation. The integration of metabolomic and microbial data provides a framework for understanding the biological mechanisms underlying variation in NDF digestibility in dairy cattle.

## Discussion

Our metagenomic and metabolomic analysis provides functional insights that extend beyond mere cataloging of microbial compositions. It highlights the functional interactions and metabolic networks responsible for different NDF digestibility phenotypes in dairy cows. By linking microbial taxonomic and functional profiles with metabolite changes and fermentation metrics, we provide an integrative framework to better understand microbial and metabolic signatures associated with NDF digestibility.

A key finding was the significant enrichment of specific bacterial taxa within the high-NDF digestibility (HI) rumen microbiome, including *Prevotella* sp., *Bacteroidaceae_bacterium*, *Succiniclasticum* sp., *Treponema* sp.*,* and the poorly characterized genus *Kiritimatiella*. Because several of the dominant taxa remained unresolved at the species level, our data are better interpreted as indicating a community-level functional shift rather than assigning a dominant mechanistic role to any single named genus. The enrichment of *Treponema*-associated taxa in the HI group is also noteworthy. Members of this lineage are frequently linked to carbohydrate-active and pectinolytic functions in the rumen and have been reported to associate with fiber-adherent microbial consortia. This ecological role is directionally consistent with the enrichment of CE8 and GH106 observed in the HI group, although our liquid-phase sampling design does not allow direct assignment of these CAZyme functions to *Treponema* itself. In this context, the co-enrichment of *Prevotella*-associated taxa together with uncultured *Bacteroidales* and *Bacteroidaceae*-related organisms, increased amino acid degradation functions, and altered metabolite profiles suggests a broader cross-feeding network linked to NDF digestibility [12, 14]. At the pathway level, the amino acid degradation signals enriched in the HI group were linked to reactions involving pyruvate, alpha-ketoglutarate, and succinyl-CoA-related steps, which are central nodes in fermentative metabolism. These associations suggest a broader community-level metabolic configuration connected to VFA production, but they do not by themselves establish a direct causal link between amino acid catabolism and fiber digestion.

A key methodological limitation of the present study is that rumen samples were obtained by esophageal tubing and then filtered through four layers of gauze before DNA extraction. As a result, the metagenomic profiles generated here mainly reflect the liquid-phase rumen microbiota and likely under-represent particle-attached fibrolytic organisms that are more abundant in the solid digesta fraction. This sampling feature may partly explain why classical fiber-adherent taxa such as *Fibrobacter* were not prominent among the differential taxa, and why the CAZyme differences observed in the HI group were more consistent with pectinolytic, debranching, and oligosaccharide-directed functions than with classical cellulolytic systems. Therefore, our conclusions should be interpreted as describing microbial and functional signatures associated primarily with the liquid-phase rumen ecosystem rather than the entire rumen fiber-adherent community.

Similarly, the enrichment of *Succiniclasticum* in HI cows is consistent with the known physiology of this genus as a succinate-utilizing rumen bacterium that converts succinate to propionate [32]. Its enrichment therefore more likely indicates greater upstream availability of succinate generated by other members of the rumen community, rather than direct fermentation of dietary carbohydrates by *Succiniclasticum* itself. This interpretation supports a directional cross-feeding relationship linked to propionate formation in the HI group. However, because acetate, propionate, and total VFAs all increased in parallel, our data support a difference in overall fermentation intensity rather than a specific shift toward propionate [33, 34]. The enrichment of *Kiritimatiella*, a poorly characterized genus predicted to encode polysaccharide-degrading enzymes [9, 10] but currently uncultured from the rumen, suggests that it may be relevant to the microbial signatures associated with higher NDF digestibility. However, its potential role is inferred from functional annotation rather than direct experimental evidence, and its specific interactions require future isolation and characterization. At the functional level, HI cows show increased abundance of genes encoding specific Glycoside Hydrolases (GH2; GH3; GH106; GH31; GH92; GH29; GH43-29) and Carbohydrate Esterases (CE8), which may contribute to polysaccharide processing in the liquid-phase rumen environment. Rather than representing a canonical cellulolytic system, these enriched families are more consistent with pectinolytic, debranching, and oligosaccharide-directed activities, including the processing of side chains and terminal residues from partially degraded plant glycans [35, 36]. These functional differences may therefore indicate enhanced utilization of soluble or partially degraded plant-derived substrates in the HI group, which may in turn support microbial fermentation and contribute to higher VFA production [37, 38]. We additionally examined canonical endo-acting cellulase- and xylanase-related families in Additional file 5. Among them, GH5 showed a higher family-level abundance in the HI group, whereas GH9, GH48, GH10, GH11, and CE1 were not significantly enriched. Several carbohydrate-binding module families also differed between groups. However, no explicit dockerin- or cellulosome-related family annotation was identified in the present dataset. Importantly, the LO group should not be interpreted merely as a reduced version of the HI phenotype. Instead, it displayed its own coordinated microbial and functional signature. Compared with the HI group, the LO group showed greater enrichment of *Methanobrevibacter* species, several glycosyltransferase-dominated CAZyme families (GT2, GT4, GT8, GT28, and GT83), and higher abundance of K00016 and K00817, indicating that the LO group displayed a distinct functional profile. However, the higher abundance of K00016 should be interpreted as a lactate-associated signal rather than direct evidence of enhanced branched-chain amino acid biosynthesis. Taken together, these patterns suggest that the LO rumen microbiome may channel a larger proportion of carbon and reducing equivalents toward microbial biosynthesis and methanogenesis rather than toward fermentative VFA output. This provides a complementary explanation for lower NDF digestibility in the LO group.

The interpretation of branched-chain amino acid metabolism at the KO level required revision after KEGG verification showed that K00016 encodes L-lactate dehydrogenase rather than ketol-acid reductoisomerase. We therefore no longer interpret K00016 as evidence of enhanced branched-chain amino acid biosynthesis in the LO group. Although pathway enrichment suggested differences in valine, leucine and isoleucine metabolism, the KO-level data in the present study were insufficient to determine the direction of branched-chain amino acid flux. By contrast, the higher abundance of K00016 in the LO group points to a lactate-associated functional signal that may be relevant to the lower NDF digestibility phenotype. Consistent with this, rumen pH tended to be lower in the LO group than in the HI group, although the difference was not statistically significant (*P* = 0.09; Table 1). This pattern is directionally consistent with the possibility that a more acidified rumen environment may be less favorable for efficient fiber degradation [39].

Although HI cows exhibited higher NDF digestibility and higher total VFA concentrations, they showed lower species-level abundance of *Methanobrevibacter*. This pattern indicates that the relationship between fiber degradation and methanogenesis in the present dataset is not simply explained by greater substrate turnover alone. Methanogenesis depends on the balance between hydrogen production and disposal, the availability of alternative electron sinks, rumen pH, substrate partitioning, and the composition of the archaeal community [33, 34]. In our data, acetate, propionate, and total VFAs all increased in parallel, whereas the acetate-to-propionate ratio was numerically similar between groups (1.73 in HI vs. 1.68 in LO), arguing against a simple shift toward propionate as the dominant alternative hydrogen sink. In addition, rumen pH tended to be lower in the LO group than in the HI group, which is directionally consistent with less favorable conditions for fiber degradation in LO rather than a pH-driven suppression of methanogens in HI. Moreover, the archaeal difference was detected at the species level, with higher abundance of several *Methanobrevibacter* species in LO, whereas no difference was observed at the phylum or genus levels. Together, these observations suggest that the methanogen signal in the present study more likely reflects a shift in archaeal community composition within a broader microbial-metabolic network than a simple direct response to fiber turnover or VFA output. Because hydrogen flux and dissolved gas pools were not directly measured, the balance among methanogenesis and alternative hydrogen sinks should be interpreted cautiously.

By partitioning the total omics-explained variance, we found that the VFA profile accounted for the largest proportional share (54.3%), which biologically validates the NDF digestibility phenotype, as VFAs are the direct terminal outputs of fiber fermentation. Conversely, the microbial composition contributed 43.0% of the explained variance, revealing the upstream driving force. It is critical to recognize that these three omics layers are highly correlated in vivo; thus, these estimated percentages represent their relative independent contributions to the model's total explanatory power (summing to 100%), rather than mutually exclusive partitions of the absolute phenotypic variance. The lower proportional explainability of the metabolome (2.7%) may occur because many small molecules are highly permeable, have rapid turnover rates, or serve as transient co-metabolic intermediates that do not stably accumulate like VFAs. This quantification strongly supports the model in which microbial enzymatic activities (CAZymes) deconstruct fiber [35, 36], fueling central fermentative pathways (KEGG modules) that produce VFAs [37–39], which are then absorbed and utilized directly by the host.

In conclusion, our multi-omics integration suggests a potential coherent microbial-metabolic network associated with high NDF digestibility. We propose a model where an enriched consortium of bacteria (*Prevotella* sp., *Succiniclasticum* sp., etc.) and the poorly characterized genus *Kiritimatiella* may contribute to polysaccharide processing via specific CAZymes.

Concurrently, amino acid degradation pathways enriched in the HI group were associated at the pathway level with central carbon metabolism and with a succinate-propionate cross-feeding configuration, although the specific intermediates and metabolic fluxes remain to be experimentally validated. This broader metabolic configuration was associated with higher overall VFA output in the HI group rather than a specific shift toward propionate. In addition, the observed metabolic environment was associated with lower methanogen abundance, although the causal basis of this relationship requires further study. Importantly, our variance partitioning analysis quantitatively demonstrates that microbial processes and VFA outputs play primary roles in governing this phenotype. These findings provide a basis for developing targeted strategies to modulate rumen microbial networks, with the goals of enhancing fiber utilization and potentially reducing methane emissions in dairy production.

## Conclusion

Overall, our findings partially support the hypothesis that variation in NDF digestibility is associated with coordinated microbial and metabolic functions rather than with the abundance of a single fibrolytic taxon. Cows with higher NDF digestibility exhibited lower species-level abundance of *Methanobrevibacter* together with stronger fermentation-related functions. However, because acetate, propionate, and total VFAs increased in parallel and the acetate-to-propionate ratio was similar between groups, this pattern should not be interpreted as evidence for a simple propionate-driven suppression of methanogenesis. Instead, it more likely reflects a broader shift in archaeal community composition and hydrogen-disposal networks that warrants direct validation. In contrast, the LO phenotype was associated with relatively greater species-level abundance of *Methanobrevibacter*, glycosyltransferase-enriched CAZyme families, a stronger lactate-associated functional signal, and higher K00817 abundance related to histidine metabolism. These contrasting signatures suggest that the HI and LO groups represent two distinct rumen microbial-metabolic configurations linked to NDF digestibility. Several bacterial lineages related to *Prevotella, Succiniclasticum*, *Treponema*, and other unresolved taxa were enriched in the high-NDF digestibility (HI) cows and were associated with alanine, aspartate, and glutamate metabolism, as well as valine, leucine, and isoleucine degradation pathways. Overall, omics-explainability analysis revealed that rumen microorganisms and rumen fermentation VFAs contributed more significantly to NDF utilization than rumen metabolomics.

However, because the present study was based on strained liquid-phase rumen samples, these findings primarily reflect the liquid-associated rumen microbiota and should not be generalized directly to the particle-attached fibrolytic community. These microbiome-associated patterns provide useful insights into strategies for modifying the rumen microbiota to enhance NDF utilization through feeding management or genetic selection.

## Supplementary Information

Additional file 1. Formula used for apparent digestibility calculation based on the acid-insoluble ashindicator method.

Additional file 2. Apparent nutrient digestibility, VFA profiles, and selected animal characteristics of the HI and LO cows.

Additional file 3. Ingredient and nutrient composition of the early-lactation basal diet used during the digestibility evaluation period.

Additional file 4. Detailed gas chromatographic method for determination of volatile fatty acid concentrations in rumen fluid.

Additional file 5. Composition of CAZymes based on the class-level and family-level enzymes.

Additional file 6. Composition of metabolic pathways based on the first-level and second-level functions in the KEGG database.

Additional file 7. Detailed results of differential KEGG Orthology based on the Wilcoxon rank-sum test.

Additional file 8. Detailed results of differential metabolites based on VIP analysis.

Additional file 9. Detailed results of differential metabolites based on KEGG enrichment analysis.

Additional file 10: Fig. S1. Taxonomic composition of the rumen microbiota at different taxonomic levels. Bar plots showing the relative abundance of the top bacterial phyla, genera, and speciesin the rumen samples of dairy cattle. The *x*-axis represents the relative abundance, and the *y*-axis indicates the samples. Taxa with low relative abundance are grouped as “Others”.

Additional file 11: Fig. S2. Differentially abundant taxa identified by LEfSe analysis. Linear discriminant analysis effect sizeidentified microbial taxa with significant differential abundance between the HI and LO groups at the species level. A shows all significantly differential taxa identified by LEfSe, and B shows the Methanobrevibacter taxa enriched in the LO group.

## Data Availability

The rumen metagenome sequences were deposited into the NCBI Sequence Read Archive (SRA) under the accession number PRJNA1180193 (https://www.ncbi.nlm.nih.gov/bioproject/PRJNA1180193).

## Abbreviations

ANOSIM: Analysis of similarity
CAZymes: Carbohydrate-active enzymes
CEs: Carbohydrate esterases
CP: Crude protein
CPM: Counts per million
DIM: Days in milk
DMI: Dry matter intake
FDR: False discovery rate
GHs: Glycoside hydrolases
GTs: Glycosyltransferases
HI: High neutral detergent fiber digestibility
HMDB: Human Metabolome Database
KEGG: Kyoto Encyclopedia of Genes and Genomes
KO: KEGG Orthology
LDA: Linear discriminant analysis
LEfSe: Linear discriminant analysis effect size
LO: Low neutral detergent fiber digestibility
NDF: Neutral detergent fiber
NST: Normalized stochasticity ratio
OPLS-DA: Orthogonal partial least squares discriminant analysis
PLs: Polysaccharide lyases
RF: Random forest
ROC: Receiver operating characteristic
TMR: Total mixed ration
VFAs: Volatile fatty acids
VIF: Variance inflation factor
VIP: Variable importance in projection
