# Supplementary material for: Multi-omics analysis reveals microbial and metabolic signatures associated with neutral detergent fiber digestibility in dairy cattle
Source: J Anim Sci Biotechnol. 2026 Nov 6;17:201. doi: 10.1186/s40104-026-01519-3 (PMC13741702; doi:10.1186/s40104-026-01519-3)
Supplement: Supplementary file 1 [file 40104_2026_1519_MOESM10_ESM.pdf]

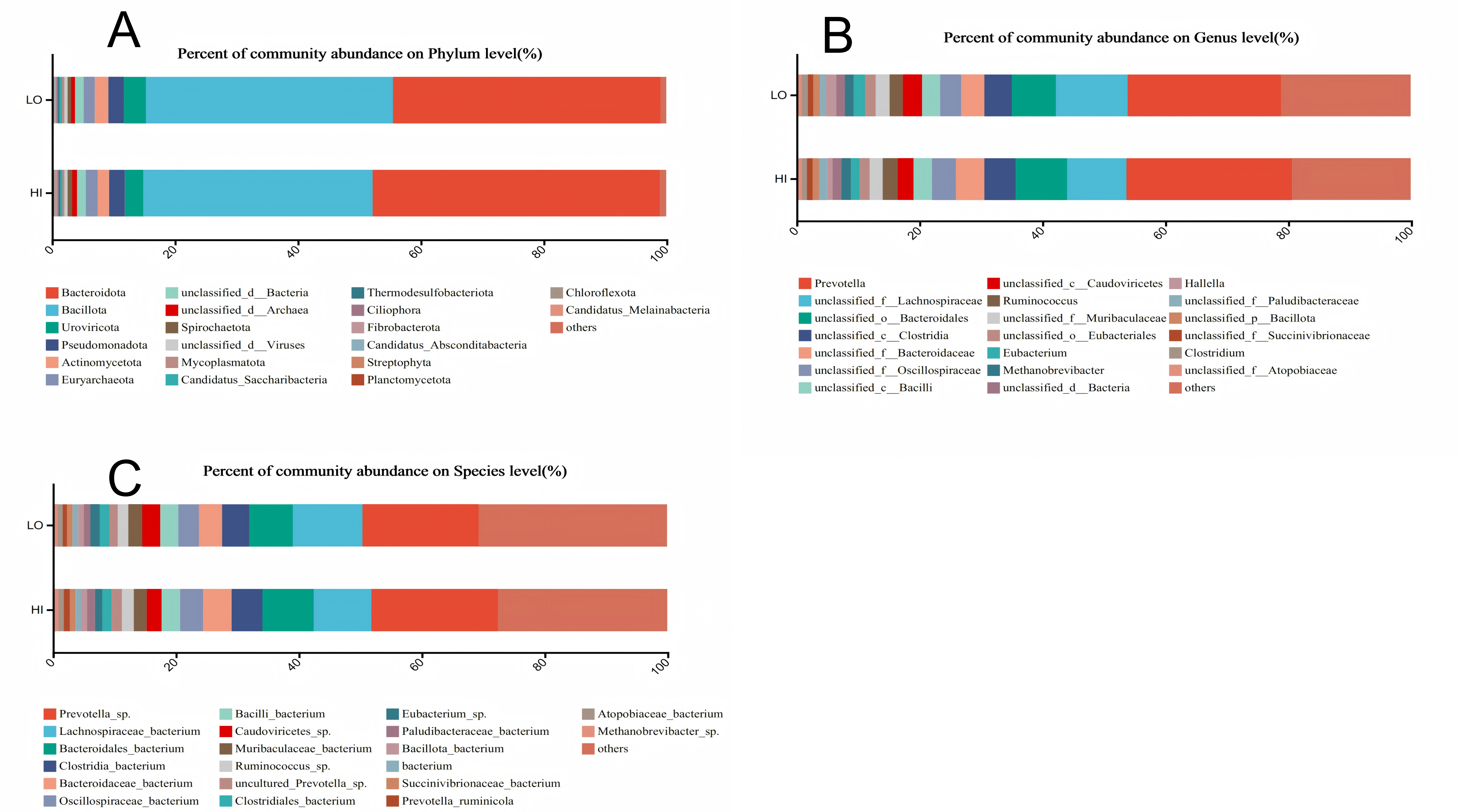

Figure S1. Taxonomic composition of the rumen microbiota at differenttaxonomic levels. Bar plots showing the relative abundance of the top dominant bacterial phyla (A)genera (B), and species (C) in the rumen samples of dairy cattle. The x-axis represents the relative abundance (%), and the y-axis indicates the samples (orgroups). Taxa with low relative abundance aregrouped as "Others"
