# Supplementary material for: Multi-omics analysis reveals microbial and metabolic signatures associated with neutral detergent fiber digestibility in dairy cattle
Source: J Anim Sci Biotechnol. 2026 Nov 6;17:201. doi: 10.1186/s40104-026-01519-3 (PMC13741702; doi:10.1186/s40104-026-01519-3)
Supplement: Supplementary file 2 [file 40104_2026_1519_MOESM11_ESM.pdf]

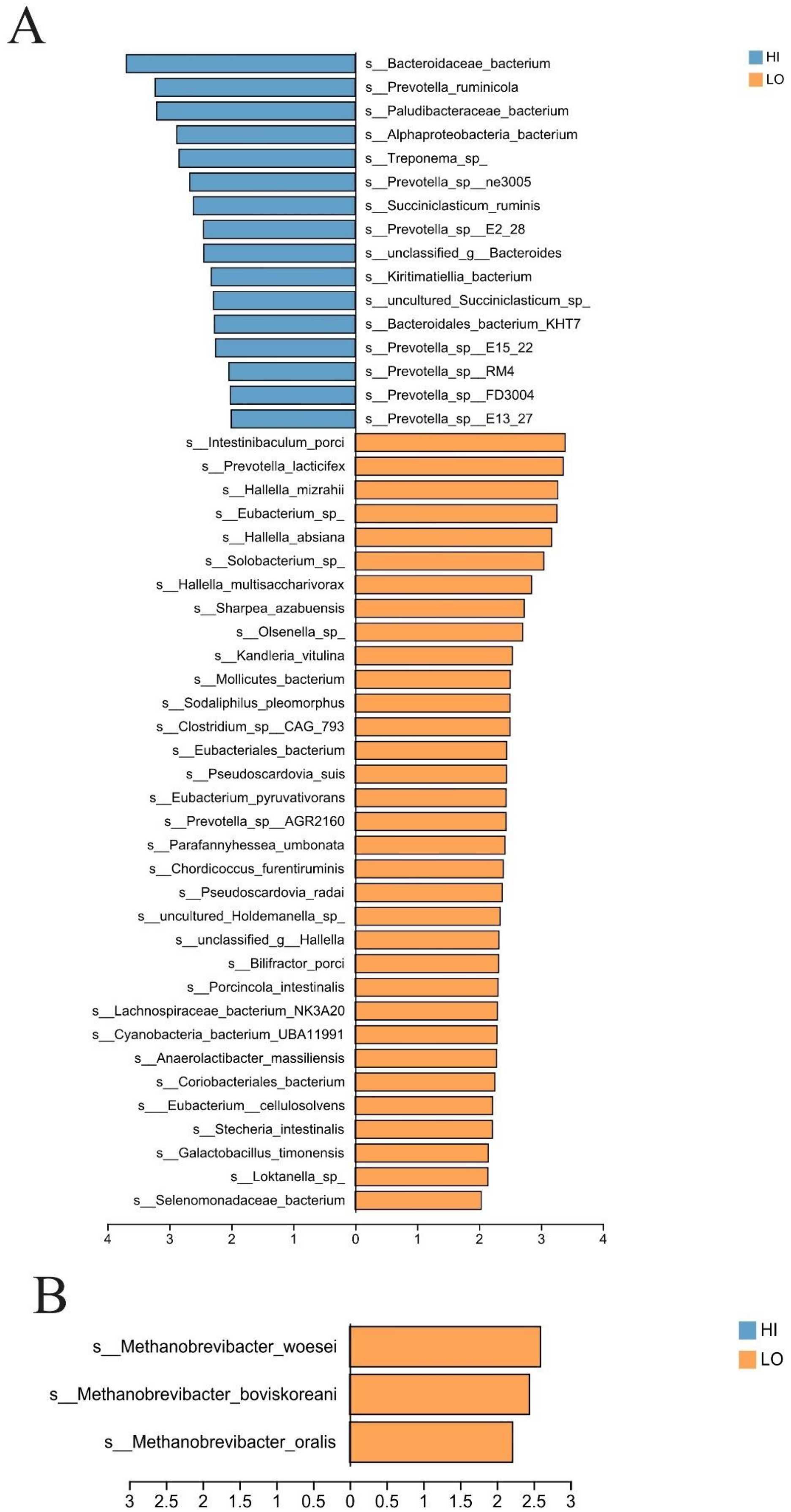

Figure S2. Differentially abundant taxa identified by LEfSe analysis. Linear discriminant analysis (LDA) effect size (LEfSe) analysis identifying the microbial taxa with significant differences in relative abundance between the HIand LOgroups (LDAscore > 3.0; $P < 0.05$). The length of the bar represents the LDA score, indicating the effect size of e ach taxon. Taxa enriched in the HIare shown in blue, and those enriched in the LOare shown in orange.
