# Supplementary material for: Multi-omics analysis reveals microbial and metabolic signatures associated with neutral detergent fiber digestibility in dairy cattle
Source: J Anim Sci Biotechnol. 2026 Nov 6;17:201. doi: 10.1186/s40104-026-01519-3 (PMC13741702; doi:10.1186/s40104-026-01519-3)
Supplement: Supplementary file 3 [file 40104_2026_1519_MOESM1_ESM.pdf]

Additional file 1. Formula used for apparent digestibility calculation based on the acid-insoluble ash (AIA) indicator method

The apparent digestibility of dietary nutrients was calculated using acid-insoluble ash (AIA) as an endogenous indicator, following the method described by Elliot et al. (1961).

Apparent digestibility (%) = [1 - (AIA in TMR / AIA in feces) × (nutrient concentration in feces / nutrient concentration in TMR)] × 100

Where AIA in TMR and AIA in feces represent the acid-insoluble ash concentrations measured in the total mixed ration and fecal samples, respectively, and nutrient concentration refers to the corresponding concentration of the target nutrient in the TMR and fecal samples.
