# Supplementary material for: Multi-omics analysis reveals microbial and metabolic signatures associated with neutral detergent fiber digestibility in dairy cattle
Source: J Anim Sci Biotechnol. 2026 Nov 6;17:201. doi: 10.1186/s40104-026-01519-3 (PMC13741702; doi:10.1186/s40104-026-01519-3)
Supplement: Supplementary file 4 [file 40104_2026_1519_MOESM3_ESM.pdf]

## Additional file 3. Ingredient and nutrient composition of the early-lactation basal diet used during the digestibility evaluation period

| Item | Early-lactating diet |
|---|---|
| **Ingredient composition, % of DM** | |
| Corn silage | 6.33 |
| Alfalfa silage | 48.96 |
| Soybean meal | 8.77 |
| Whole cottonseed | 4.61 |
| Cottonseed meal | 1.23 |
| Canola meal | 1.83 |
| Molasses | 5.31 |
| Beet pulp | 2.65 |
| DDGS | 1.43 |
| DDG | 1.43 |
| Fat powder | 13.26 |
| Calcium salts of fatty acids | 0.98 |
| Rumen-protected nicotinic acid | 0.31 |
| Feed additive | 1.43 |
| Limestone | 0.21 |
| Mineral and vitamin premix | 1.26 |
| **Chemical composition** | |
| DM, % as-fed basis | 47.28 |
| CP, % of DM | 17.17 |
| NDF, % of DM | 31.05 |
| ADF, % of DM | 16.59 |
| Starch, % of DM | 29.38 |
| EE, % of DM | 4.64 |
| Ash, % of DM | 7.83 |

All cows were fed the same early-lactation basal diet during the digestibility evaluation period. Because the animals were group-housed, individual dry matter intake (DMI) was not recorded. The mineral and vitamin premix for the early-lactation diet contained (DM basis): 480 mg/kg Cu, 28 mg/kg I, 1,600 mg/kg Mn, 1,800 mg/kg Zn, 13 mg/kg Se, 28 mg/kg Co, 210,000 IU/kg vitamin A, 70,000 IU/kg vitamin D, and 5,600 mg/kg vitamin E.
