# Supplementary material for: Multi-omics analysis reveals microbial and metabolic signatures associated with neutral detergent fiber digestibility in dairy cattle
Source: J Anim Sci Biotechnol. 2026 Nov 6;17:201. doi: 10.1186/s40104-026-01519-3 (PMC13741702; doi:10.1186/s40104-026-01519-3)
Supplement: Supplementary file 5 [file 40104_2026_1519_MOESM4_ESM.pdf]

Additional file 4. Detailed gas chromatographic method for determination of volatile fatty acid concentrations in rumen fluid.

Method basis

Rumen volatile fatty acid (VFA) concentrations were determined using an Agilent 7890B-7000D gas chromatography platform. Sample preparation and chromatographic separation were based on the direct gas chromatographic method of Erwin et al. (1961) and an optimized laboratory protocol adapted from Cao et al. (2008).

Analytes and standards

The following VFAs were quantified: acetate, propionate, isobutyrate, butyrate, isovalerate, and valerate. 2-Ethylbutyric acid was used as the internal standard. Metaphosphoric acid was used for deproteinization.

Preparation of deproteinizing solution

A 25% (w/v) metaphosphoric acid solution containing 2-ethylbutyric acid (2 g/L) was prepared and used as the deproteinizing solution and internal-standard solution.

Preparation of mixed standard stock solution

A mixed VFA standard stock solution containing acetate, propionate, isobutyrate, butyrate, isovalerate, and valerate was prepared. Calibration standards were generated from this stock solution by serial dilution with the internal-standard metaphosphoric acid solution to obtain five concentration levels for quantitative analysis.

Sample preparation

Rumen fluid samples were first centrifuged at 5,400 rpm for 10 min to remove feed particles and other debris. Then, 1.0 mL of the clarified supernatant was transferred into a 1.5 mL centrifuge tube and mixed with 0.2 mL of 25% metaphosphoric acid solution containing 2-ethylbutyric acid as the internal standard. After mixing, the samples were incubated in an ice-water bath for at least 30 min for protein precipitation. The samples were then centrifuged again at 10,000 rpm for 10 min, and the resulting supernatant was used for chromatographic analysis.

Chromatographic conditions

Chromatographic separation was carried out using an HP-INNOWax capillary column (30.0 m × 320 um × 0.5 µm). Nitrogen was used as the carrier gas. The split ratio was 40:1, and the injection volume was 0.4 µL. The injector temperature was set at 220 C. The oven temperature program was 120 C for 3 min, increased at 10 C/min to 180 C, and then held for 1 min.

Quantification

Quantification was performed using the internal-standard method with 2-ethylbutyric acid. Calibration curves were generated from the five-level mixed standard solutions, and VFA concentrations in unknown samples were calculated from the peak-area ratio between each analyte and the internal standard. Total VFA concentration was calculated as the sum of the measured individual VFAs

References to add to the manuscript reference list

Erwin E S, Marco G J and Emery E M. Volatile Fatty Acid Analyses of Blood and Rumen Fluid by Gas Chromatography[J]. Journal of Dairy Science, 1961, 44(9): 1768-1771. https://doi.org/10.3168/jds.S0022-0302(61)89956-6.

Cao Z J, Li S L, Xing J J, et al. Effects of maize grain and lucerne particle size on ruminal fermentation, digestibility and performance of cows in midlactation[J]. Journal of Animal Physiology and Animal Nutrition, 2008, 92(2): 157-167. https://doi.org/10.1111/j.1439-0396.2007.00721.x.
